# The Involvement of Resolvins in Pathological Mechanisms of Periodontal Disease Associated with Type 2 Diabetes: A Narrative Review

**DOI:** 10.3390/ijms252312784

**Published:** 2024-11-28

**Authors:** Larisa Ghemiș, Ancuta Goriuc, Raluca Jipu, Liliana Georgeta Foia, Ionut Luchian

**Affiliations:** 1 Department of General and Oral Biochemistry, Faculty of Dental Medicine, “Grigore T. Popa” University of Medicine and Pharmacy Iaşi, 700115 Iași, Romania; 2 Department of Morpho-Functional Sciences, Faculty of Medicine, “Grigore T. Popa” University of Medicine and Pharmacy Iași, 700115 Iași, Romania; 3 Department of Periodontology, Faculty of Dental Medicine, “Grigore T. Popa” University of Medicine and Pharmacy Iaşi, 700115 Iași, Romania

**Keywords:** resolvins, periodontal disease, type 2 diabetes, inflammation

## Abstract

Resolvins are specialized pro-resolving mediators (SPMs) derived from omega-3 fatty acids that play a critical role in resolving inflammation and restoring tissues to a state of health after an immune response. Their role in chronic inflammatory conditions highlights their importance in maintaining a balance between an effective immune response and the resolution of inflammation to prevent tissue damage. Periodontal disease is a chronic inflammatory condition affecting the tissues surrounding the teeth, leading to gum damage and bone loss. Chronic inflammation in periodontal disease can exacerbate systemic inflammation and influence other conditions, such as diabetes. There is a bidirectional relationship between diabetes and periodontal disease, as both are characterized by chronic inflammation and exacerbate systemic and oral health complications. This narrative review aims to synthesize the current knowledge on how resolvins influence inflammatory pathways and the tissue repair mechanism in periodontal disease in patients with type 2 diabetes. Furthermore, this review serves as a foundation for developing targeted therapeutic strategies, addressing the pressing need for effective treatments that consider both systemic and oral health outcomes.

## 1. Introduction

Periodontitis is a condition affecting the periodontal tissues (alveolar bone, gingiva, cementum, and periodontal ligament), caused by various factors that lead to an inadequate inflammatory response [1,2]. It is a chronic condition, predominantly found in adults, characterized by periods of activity, and if left untreated, it can lead to tooth loss [3,4]. A key factor in maintaining oral health is the oral microbiome, which, once it becomes dysbiotic, can directly contribute to tissue destruction [5]. In this context, the bacteria present in the biofilm can trigger an exaggerated inflammatory response which, if unresolved, contributes to the development of periodontitis [6]. One of the Gram-negative anaerobic bacteria present in the biofilm, and considered the main pathogen involved in pulp infection and periodontitis, is *Porphyromonas gingivalis* (*P. gingivalis*) [7].

Foreign antigens can initiate a physiological inflammatory response, which is crucial for maintaining the body’s health. Inflammation is associated with changes in the function of affected tissues, the presence of an immune cell infiltrate, and the increased local production of cytokines and chemokines [8]. Mechanical stimulation through chewing, along with the commensal microbiota, induces a limited immune response, represented by the infiltration of innate lymphoid cells and Th17 cells into the gingival tissue, as well as neutrophils into the gingival sulcus [9]. With the onset of dental plaque dysbiosis, the host’s immune response becomes over-activated, leading to the secretion of pro-inflammatory cytokines such as IL-1, IL-6, and TNF-α [4,9,10]. These cytokines promote the recruitment, activation, and differentiation of immune cells, as well as the activation of osteoclasts responsible for tissue destruction [9]. Bone loss associated with periodontal disease may be linked to the receptor activator of the nuclear factor-kappa B (RANK)-RANK ligand (RANKL)-osteoprotegerin (OPG) axis [11]. Failure to resolve acute inflammation and restore tissue homeostasis leads to chronic inflammation, along with bone and extracellular matrix destruction, resulting in scar formation and fibrosis [12].

The inflammatory state present in periodontitis can elicit a systemic response, manifesting as low-grade systemic inflammation [2]. The presence of periodontal disease in a patient may induce an increase in the gene expression levels of CRP, IL-6, and TNF-α, which, in turn, can exacerbate this inflammatory state [10]. A possible cause of the systemic inflammation associated with periodontitis is the hematogenous dissemination of both the inflammatory mediators produced in excess in the periodontal tissues and periodontal bacteria [13]. With the destruction of the periodontal pocket epithelium, bacteria, along with endo- and exotoxins, can enter the bloodstream and initiate a systemic inflammatory response. Furthermore, the detection of periodontal pathogens in other organs has led to the association of periodontitis with various systemic disorders [14]. Recent studies and meta-analyses demonstrate a direct association between periodontitis and several systemic diseases, such as obesity, type 2 diabetes, and non-alcoholic fatty liver disease, conditions that are also associated with low-grade chronic inflammation [15,16,17]. In this inflammatory context, a novel class of mediators, known as resolvins, are involved. Despite advances in understanding their pathological mechanism, the role of resolvins, specialized pro-resolving mediators with anti-inflammatory and tissue-repairing proprieties, remains underexplored in this dual context. This narrative review aims to explore the roles of resolvins in modulating chronic inflammation associated with both periodontal disease and type 2 diabetes, highlighting the link between these two significant pathologies [18]. By integrating existing evidence, this review identifies knowledge gaps and guides future research, contributing to the development of targeted therapies that improve both systemic and oral health outcomes in patients with periodontal disease associated with type 2 diabetes.

## 2. Methodology

The purpose of this narrative review is to explore the involvement of resolvins in the pathological mechanisms linking periodontal disease and type 2 diabetes. A literature search was performed across major databases, including PubMed, Google Scholar, Web of Science, and Scopus. Keywords such as “resolvins”, “specialized pro-resolving mediators”, “periodontal disease”, “type 2 diabetes”, and “inflammation” were used in various combinations to identify relevant studies. The search focused on articles published in the last 5–10 years, prioritizing peer-reviewed experimental, clinical, and in vitro studies, while foundational research was included to provide background information. Only studies written in English were considered.

Inclusion criteria focused on studies investigating the role of resolvins in inflammation modulation within the context of two major pathologies: periodontal disease and type 2 diabetes. Non-peer-reviewed articles, studies lacking sufficient methodological detail, and articles published in a language other than English were excluded. Relevant information from selected articles was organized into sections using the IMRAD structure (Introduction, Methods, Results, and Discussion), along with additional chapters that review the dual relationship between periodontal disease and type 2 diabetes, the biological roles of resolvins, and their anti-inflammatory properties in both periodontal disease and type 2 diabetes. This methodology ensures a comprehensive and structured exploration of the topic while allowing the identification of research gaps and future research directions.

## 3. Periodontal Disease and Type 2 Diabetes

Diabetes mellitus is a complex, chronic, and progressive metabolic disorder with a significant impact on health. In 2021, approximately 537 million cases were diagnosed, and estimates predict an increase to 643 million cases by 2030 and 783 million by 2045 [19,20]. Type 2 diabetes (T2D) accounts for over 90% of all diabetes cases. Its main characteristic is peripheral insulin resistance, accompanied by dysfunction and eventual loss of pancreatic beta cells, phenomena responsible for the chronic hyperglycemia specific to this disease [21,22,23].

**Figure 1:**
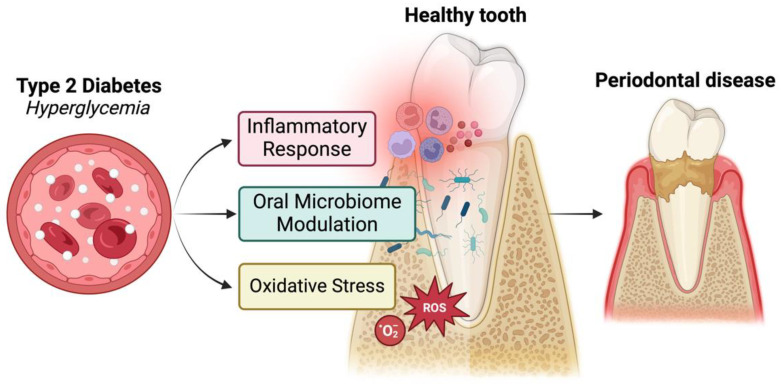
The mechanisms underlying the relationship between type 2 diabetes and periodontal disease: increased inflammatory response, oral microbiome modulation, and oxidative stress. Created in BioRender. Ghemis, L. (2024) BioRender.com/w90x393.

Diabetes mellitus is associated with both macrovascular complications, such as stroke and coronary heart disease, and microvascular complications, including neuropathy, nephropathy, and retinopathy [19]. Diabetes mellitus is a major risk factor for the development of periodontitis, which is considered the sixth complication of diabetes mellitus [24,25]. Type 2 diabetes can worsen periodontitis, but this relationship appears to be bidirectional. Periodontitis, in turn, can negatively affect glycemic control and increase the risk of chronic complications of diabetes [24]. The mechanisms underlying this biological link include microbiota dysbiosis, inflammatory response, and oxidative stress, and are illustrated in Figure 1 [26].

Obesity and type 2 diabetes are considered risk factors and even aggravating factors for chronic periodontitis [27,28]. Diabetes triggers immune and inflammatory responses in the periodontal tissues, increasing the risk of periodontitis. The subsequent secretion of cytokines and amplification of oxidative damage, along with disrupted receptor-mediated signaling, accelerate both the destruction of periodontal tissues and bone resorption, worsening periodontitis [29]. Furthermore, wound healing in periodontal tissues is impaired in type 2 diabetes patients. One of the mechanisms driving this impairment is excessive systemic inflammation, alongside a deficient and imbalanced cellular immune response, observed in neutrophils as well as in Th1, Th2, and Th17 cells [4]. Imbalanced oxidative stress is another critical link between type 2 diabetes and chronic periodontitis, as both individually produce oxidative damage. Chronic periodontitis affects local gingival tissues, while type 2 diabetes has systemic effects, reducing the body’s antioxidant capacity. The presence of both type 2 diabetes and chronic periodontitis exacerbates oxidative stress both locally and systemically, leading to increased gingival tissue destruction [30].

The periodontal microbiome is also influenced by the chronic inflammation present in type 2 diabetes. It can significantly alter the salivary and subgingival microbiome and increase the inflammatory burden on periodontal tissues [13,31]. Additionally, the presence of diabetes may influence the host’s response to pathogenic microorganisms [32]. Moreover, non-surgical periodontal treatment improves and stabilizes the oral microbiota in patients with periodontitis and diabetes, and positively impacts their glycemic control [33]. Non-surgical periodontal treatment over a six-month period lowers glycated hemoglobin (HbA1c) levels and reduces the levels of pro-inflammatory mediators [10,34]. Therefore, there is a bidirectional relationship between type 2 diabetes and periodontal disease, driven by the low-grade chronic inflammation present in both conditions [10]. The inflammatory nature of periodontitis can aggravate the systemic inflammation in type 2 diabetes by increasing the levels of pro-inflammatory mediators, such as IL-1, IL-6, and TNF-α, through the activation of inflammatory cascades that lead to the secretion of these cytokines. These mediators are directly involved in the development of insulin resistance and, subsequently, type 2 diabetes [10,35]. Recently, a new class of mediators has been described that directly participates in mediating this inflammation. Specifically, the persistence of the inflammatory syndrome may be attributed to the deficiency of these mediators, known as specialized pro-resolving mediators (SPMs), with resolvins being among the most important [18].

## 4. Resolvins and Their Role in Inflammation

### 4.1. The Origin and Classification of Resolvins

During inflammation, a variety of lipid molecules are generated through the metabolism of membrane phospholipids in cells involved in the immune response [36]. Resolvins (Rvs) are part of a new class of endogenous lipid mediators (LMs) known as specialized pro-resolving mediators (SPMs), which are involved in the resolution of inflammation, along with maresins, protectins, and lipoxins [37,38]. These are metabolites of omega-3 polyunsaturated fatty acids (PUFAs), and there are two classes of resolvins based on the precursor polyunsaturated fatty acid [39]. Resolvins of the D-series (RvD1-RvD6) are derived from docosahexaenoic acid (DHA) through the action of the enzyme 15-lipoxygenase (15-LOX), while resolvins of the E-series (RvE1-RvE4) are derived from eicosapentaenoic acid (EPA) through the action of the enzymes leukocyte 5-LOX (resulting in RvE1 and RvE2) and 15-LOX (resulting in RvE3 and RvE4) [40,41]. Through the action of microbial enzyme cytochrome P450 and acetylated COX-2 on EPA in the presence of aspirin, 18S- or 18R-hydroxyeicosapentaenoic acid (18R- or 18S-HEPE) is generated, which is then catalyzed by 5-LOX to produce 18S-RvE1, a bioactive isomer of RvE1 [37,41]. Similarly, aspirin’s ability to modify COX-2 activity leads to the generation of AT-RvD1-4 [42].

### 4.2. Resolvins and Their Role in Acute Inflammation

**Figure 2:**
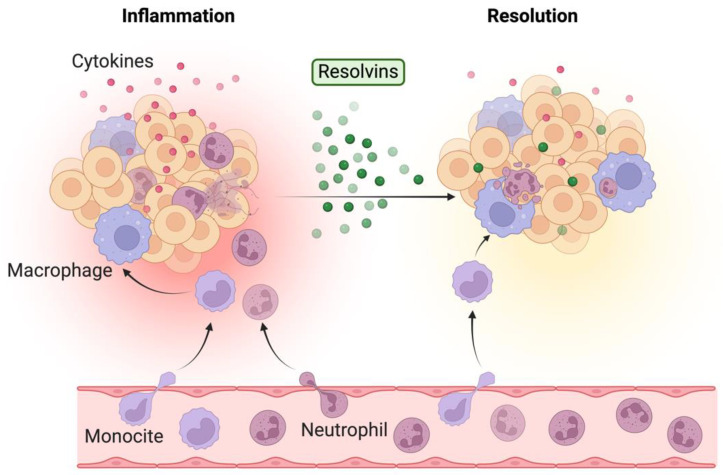
The contribution of resolvins to the modulation of inflammatory responses: resolvins decrease neutrophil infiltration, stimulate macrophage phagocytosis of apoptotic neutrophils, reduce the release of NETs by neutrophils, and lower the expression and secretion of cytokines. Created in BioRender. Ghemis, L. (2024) BioRender.com/q63g293.

The resolution process of acute inflammation is complex and involves a series of steps, such as the elimination of the inciting stimulus, the catabolism of pro-inflammatory mediators, the inhibition of leukocyte adhesion, and the stimulation of phagocytosis of apoptotic cells, leading to the clearance of the lesion, as illustrated in Figure 2 [43].

The ability of resolvins to promote the resolution of acute inflammation stems from their interaction with high-affinity surface membrane receptors, such as the G protein-coupled receptors (GPCRs) expressed on polymorphonuclear leukocytes [36]. RvD1 limits neutrophil infiltration by binding to two receptors, ALX and GPR32 [44]. A potential mechanism through which ALX/FPR2 activation (lipoxin A4 receptor/formyl peptide receptor 2) reduces neutrophil infiltration into alveolar tissue may involve the inhibition of CXCL2 expression on resident macrophages [45]. The overexpression of these two receptors also enhances macrophage phagocytosis of apoptotic PMNs and zymosan [44]. This effect is also observed in transgenic mice hGPR32_myc_Tg×Fpr2^−/−^×Apoe^−/−^, where aspirin-triggered resolvin D1 (AT-RvD1) binding to GPR32 stimulates macrophage phagocytosis. Both ALX/FPR2 and GPR32 receptors can also be activated by RvD3 and RvD5 [46].

Another important action of resolvins, impacting the resolution of inflammation, is their ability to reduce cytokine expression and secretion. This property of resolvins has been demonstrated in relation to angiotensin II, where RvD1 prevented the pro-inflammatory effects of Ang II, as seen in the increased levels of cytokines such as IL-6, MCP-1 (Monocyte Chemoattractant Protein-1), and TNF-α [47]. Enhanced macrophage phagocytosis is also observed following the binding of RvD2 to its receptor GRP18. In this case, the mechanism involves the activation of intracellular signaling pathways through the phosphorylation of CREB, ERK1/2, and STAT3, observed only in wild-type (WT) macrophages [48]. RvD4 can influence neutrophil function by reducing the release of neutrophil extracellular traps (NETs), with an impact on thrombosis development [49,50].

Resolvin E1 can mediate immune cell infiltration by acting on the G protein-coupled receptor ChemR23 (chemerin receptor 23), inhibiting the canonical NF-κB/Ccl5 pathway [51]. The primary cells expressing ChemR23 include dendritic cells, monocytes, macrophages, certain Natural Killer (NK) cells, as well as endothelial cells and adipocytes. ChemR23 activation also enhances macrophage phagocytic activity and may even induce their anti-inflammatory phenotype [52]. RvE1 can also bind to the BLT1 receptor, a high-affinity receptor for LTB4, expressed on osteoclasts and PMNs. The anti-inflammatory actions of RvE1 may result from its partial agonist effect on the BLT1 receptor, limiting LTB4-mediated leukocyte activation and infiltration [43].

## 5. Resolvins and Periodontal Disease

The inflammatory etiology of periodontal disease is multifactorial, initiated by dysbiosis but sustained and intensified by chronic inflammation [38]. This chronic inflammatory state results from the failure of the innate immune system to resolve acute inflammation. Resolvins are compounds with pro-resolving and anti-inflammatory proprieties capable of aiding in tissue regeneration and the elimination of microorganisms in periodontitis [53]. The roles and mechanism of action of resolvins in periodontal disease are summarized in Table 1.

### 5.1. D-Series Resolvins and Periodontal Disease

A study that analyzed the action of RvD1_n-3 DPA_ on oral epithelial cells, previously stimulated with TNF-α to induce an inflammatory state, concluded that this resolvin not only can prevent inflammation but also reverse it. This result is based on the action of RvD1_n-3 DPA_ on the NF-κB signaling pathway, particularly on the NF-κB p65 component, where it can reverse the TNF-α-induced translocation [54]. Besides its anti-inflammatory effect, RvD1 can stimulate osteoblast differentiation, thus having a bone regenerative effect. RvD1 can reduce osteoclast activity by decreasing the RANKL/OPG ratio, following an increase in OPG secreted by osteoblasts [55]. These results are supported by Klein’s study, which observes that during prolonged inflammatory states, osteoclastogenesis is directly inhibited by RvD1 through a decrease in RANK expression [68].

The periodontal regeneration process is complex and involves several factors, including periodontal ligament fibroblasts (PDLFs). These cells express both RvD1 receptors, GPR32 and ALX/FPR2. The pro-inflammatory cytokine IL-1β can inhibit PDLFs’ ability to proliferate and heal wounds while stimulating the production of MMPs and other pro-inflammatory cytokines. Zarrough’s study demonstrates that RvD1 can reverse and inhibit these actions of IL-1β, further proving the anti-inflammatory and pro-regenerative properties of this resolvin [56]. Moreover, RvD1 can reduce IL-1β expression in periodontal ligament cells (PDLCs) with a pro-inflammatory phenotype induced by hypoxia, and can promote the formation of calcium nodules in these cells by influencing the p38 MAPK signaling pathway [57]. Therefore, RvD1 levels in the gingival crevicular fluid can be considered a marker correlating with the healing stage following periodontal treatment, which is useful in identifying cases where treatment needs to be supplemented [69].

In endodontic infections, resolvins RvD2 and RvE1 have been detected in the periapical tissues of teeth, and treatment with N-acetylcysteine has been shown to increase the levels of these two resolvins after 14 days of treatment [70]. RvD2 can reduce the size of periapical lesions and improve the mineralization of root canal apices by decreasing MPO (myeloperoxidase) activity and, consequently, reducing the influx of neutrophils, monocytes, and leukocytes [58,59]. Root canal treatment with RvD2 leads to increased expression of GPR18 receptors both inside and outside the canals, with dental pulp cells expressing higher mRNA levels of GPR18 after three days of treatment with 10 nM RvD2 [58]. Additionally, by directly acting on GPR18 expressed on pulp tissue cells, RvD2 induces reparative dentin formation and can induce the proliferation of dental pulp stem cells (DPSCs) [60]. RvD2 treatment also reduces the frequency of Foxp3^+^CD4^+^ Treg cells and CD4^+^ T cells, which is associated with the tissue homeostasis of immune cells and pro-inflammatory cytokines. The RANKL/OPG axis, formed by RANKL expression on CD4^+^ T cells and OPG, is directly correlated with bone homeostasis, and an increased RANKL/OPG ratio is associated with chronic periodontitis and the progression of apical chronic periodontitis [61,71,72]. RvD2 can decrease RANKL expression and increase OPG expression, thus preventing alveolar bone loss [61]. Furthermore, through its direct action on osteoblasts, RvD2 can mitigate the effect of elevated testosterone levels in increasing the RANKL/OPG ratio, reversing inflammation-induced bone loss [73].

The pro-resolving effect of RvE3 is evident in relation to Escherichia coli-induced peritonitis, where RvE3 administration leads to increased phagocytosis of bacteria and neutrophils and reduces pro-inflammatory mediators such as metalloproteinases (MMP-2, MMP-9), chemokines, and cytokines, thus reducing inflammation [74]. Resolvin D5 has a similar effect on E. coli infection, reducing the production of inflammatory mediators and improving bacterial phagocytosis [75]. Given the inflammatory nature of periodontal disease, further studies are needed to evaluate the effect of resolvin E3 on the inflammatory microenvironment and dysbiosis present in this pathology.

### 5.2. E-Series Resolvins and Periodontal Disease

Resolvin E1 has a similar action to RvD1, as both regulate mineralization, proliferation, and gene expression in cementoblasts, but influence tissue degradation differently [76]. RvE1 reduces bone resorption by inhibiting the growth and differentiation of osteoclasts and through direct action on the bone, favorably promoting the RANKL/OPG and receptor activator of NF-κB ligand/OPG ratios [62,63,64]. Additionally, treatment with RvE1 of mesenchymal stem cells (MSCs) stimulated with LPS (lipopolysaccharide) to induce an inflammatory environment induced the formation of calcified deposits and promoted osteogenic differentiation, thus enhancing bone regeneration [77]. The improvement of bone regeneration is also demonstrated in Alrumaih’s study on Wistar rats with calvarial defects, which observed this effect of RvE1 in combination with adjunct bovine bone graft [78].

Besides bone regeneration, RvE1 promotes inflammation resolution by reducing CRP and IL-1β [65]. The inhibition of soluble epoxide hydrolase (sEH), responsible for the inactivation of epoxyeicosatrienoic acids (EETs) and epoxy fatty acids (EpFAs), leads to increased levels of RvE1 and RvE2, along with the overexpression of the ALX/FPR2, ChemR23, and LTB4R1 receptors, and is associated with promoting the pro-resolving phenotype of macrophages [66]. RvE1 induces dental pulp regeneration, reduces the severity of infection, and modulates inflammation resolution [79].

Localized Aggressive Periodontitis (LAP) is a rapidly progressing form of inflammatory periodontal disease characterized by neutrophil-mediated tissue destruction. In this context, RvE1 can bind to human neutrophils, and exposure to RvE1 can modify their abnormal activity [80]. Moreover, RvE1 restores the phagocytic activity of macrophages in LAP subjects [67]. There are conflicting results, as Damgaard’s study did not identify any impact of RvE1 on the production of cytokines (TNF-α and IL-6) and chemokines (CXCL8 and CCL2) by neutrophils in healthy patients and those with LAP. However, recent studies present solid evidence supporting the anti-inflammatory activity of RvE1 [81]. Additionally, RvE1 administration has been shown to inhibit effector T cells and has a protective effect on regulatory T cells (Tregs), a mechanism that contributes to reducing inflammation [82].

The anti-inflammatory capacity of RvE1 is complemented by its proven antimicrobial activity against Aggregatibacter actinomycetemcomitans (A. actinomycetemcomitans), with an MIC of 1.25 μg/mL, as RvE1 significantly slows bacterial growth [83]. Changes in the subgingival microbiota are determined by periodontal inflammation, where resolvins play a major role in the disease’s progression [84]. Treatment with RvE1 reduced inflammation by reversing the expression of inflammatory genes and decreasing osteoclast density. Consequently, the subgingival microbiota, in the case of the rats on which the experiment was conducted, underwent significant changes, with bacterial growth being inhibited [85]. Scientific evidence on the role of resolvins E3 and E4 in mediating inflammation and their contribution to resolving periodontal inflammation is limited. Fukada’s study demonstrates a greater anti-inflammatory activity of 18-Deoxy-resolvin E3 compared to resolvin E3, but further studies are needed to understand the role of these resolvins in periodontal disease [86].

## 6. Resolvins and Type 2 Diabetes

The inflammatory component of type 2 diabetes may stem from a failure in the resolution of inflammation, which is closely associated with insulin resistance and obesity. Although the exact mechanisms are not fully understood, the unresolved inflammation observed in the adipose tissue of obese patients has been correlated with a deficiency in anti-inflammatory mediators [87]. An abnormal immune response can lead to the chronicization of inflammation and subsequently to the development of chronic inflammatory diseases, such as diabetes mellitus and cardiovascular disease [88,89,90].

In uncontrolled type 2 diabetes, the expression of ERV-1, the receptor for RvE1 in neutrophils, is altered. This can be corrected by exogenous supplementation with RvE1, which activates pro-resolving signals and boosts phagocytosis in type 2 diabetes [91]. Treatment with RvE1 can induce dose-dependent changes in the expression of inflammation-related genes in diabetic neutrophils [92]. Additionally, it improves insulin sensitivity by activating the PI3K/Akt signaling pathways, leading to increased glucose uptake in adipocytes [93]. As for RvD1, its administration promotes the resolution of peritonitis by enhancing phagocytosis in diabetic macrophages and improves wound healing by decreasing macrophage and apoptotic cell accumulation at the wound site [94]. Additionally, RvD1 treatment directly prevents the development of type 2 diabetes in mice and suppresses oxidative stress while stimulating the production of lipoxin A4 (LXA4) [95]. While high LXA4 levels are strongly associated with a lower risk of developing type 2 diabetes, a recent study links increased levels of RvD1 and RvD2 to a higher risk of developing this condition, highlighting the need for further research to clarify their role in its progression [96,97].

A significant complication of type 2 diabetes is cardiovascular damage, including atherosclerotic cardiovascular disease and diabetic cardiomyopathy [98]. In atherosclerosis, resolvins play a crucial role in reducing inflammation within plaques. They promote the resolution of inflammation by enhancing the clearance of apoptotic cells and debris by macrophages [99]. This action helps to stabilize atherosclerotic plaques, potentially reducing the risk of plaque rupture and subsequent cardiovascular events. Additionally, resolvins have been shown to decrease the recruitment of inflammatory cells to the arterial wall and inhibit the production of pro-inflammatory cytokines [100]. Regarding heart failure, resolvins may help prevent disease progression through multiple mechanisms. They have been found to reduce cardiac fibrosis, a key factor in the development of heart failure. Resolvins also improve cardiac function by modulating the immune response, reducing oxidative stress, and promoting the survival of cardiomyocytes [99]. These effects collectively contribute to maintaining cardiac structure and function, potentially slowing or halting the progression of heart failure.

Non-alcoholic fatty liver disease (NAFLD) is another condition closely linked to type 2 diabetes, with a well-established bidirectional relationship between them. The liver plays a central role in the development of insulin resistance, a key factor contributing to the onset of both diseases. Type 2 diabetes increases the risk of NAFLD progression, while the presence of NAFLD is associated with a higher risk of type 2 diabetes complications [101]. RvD1 may reduce oxidative stress, suppress cytokine production, and inhibit macrophage infiltration in MCD diet-induced non-alcoholic steatohepatitis (NASH). RvD1, by reducing inflammation, fibrosis, and steatosis, emerges as a promising hepatoprotective therapeutic agent [102]. Resolvins represent a novel and promising therapeutic approach also for managing type 2 diabetes. However, clinical trials assessing their role in targeting type 2 diabetes-related inflammation are limited, highlighting the need for further studies to clarify their efficacy and safety.

## 7. Results and Discussion

**Figure 3:**
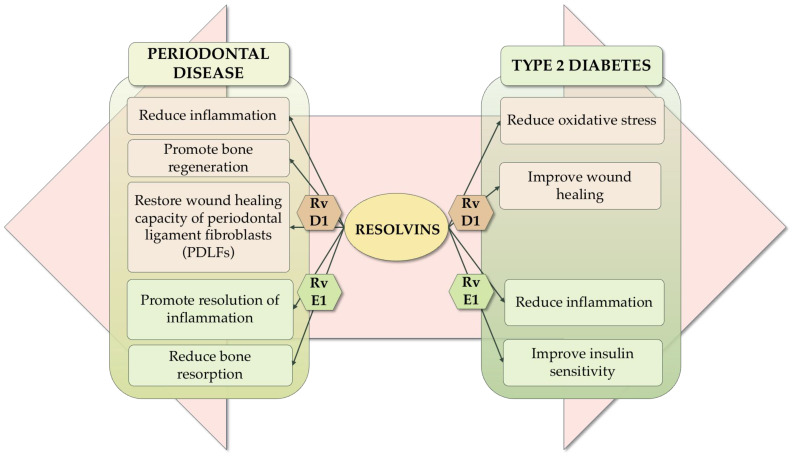
The interrelationship between resolvins, periodontal disease, and type 2 diabetes.

The interrelationship between resolvins, periodontal disease, and type 2 diabetes is complex and tied to the intricacies of immune function. Both type 2 diabetes and periodontal disease have a major inflammatory component. Resolvins, as bioactive lipid mediators with a key role in modulating inflammation, can influence the inflammatory process in both conditions as well as the progression of the disease, as illustrated in Figure 3.

An important component of periodontal disease is oral dysbiosis, which can be exacerbated by the presence of type 2 diabetes, also affecting the host’s response to foreign microorganisms [32]. Diabetic mice are more susceptible to periodontitis due to impaired neutrophil phagocytosis and the clearance of *P. gingivalis*, leading to severe tissue damage. RvE1 improves neutrophil function and pathogen clearance by dampening the phosphorylation of neutrophil signaling pathways, such as Akt and MAPK, thereby reducing inflammation. Emerging evidence indicates that persistent inflammation, driven by a deficiency of lipoxins and resolvins, is an underlying factor in both periodontitis and type 2 diabetes [18]. In periodontitis, RvE1 promotes the resolution of inflammation and reduces bone resorption, while in type 2 diabetes, it enhances insulin sensitivity [62,63,64,65,66,67,93]. RvD1 prevents and reverses inflammation in periodontitis by modulating the NF-κB signaling pathway, promotes bone regeneration, and restores the proliferation and wound healing capacity of periodontal ligament fibroblasts. In type 2 diabetes, RvD1 reduces oxidative stress and improves wound healing [54,55,56,94,95].

These connections underscore the potential of resolvins as a comprehensive therapeutic approach for managing both periodontitis and type 2 diabetes. The main reason why future studies are important is the significant impact of diabetes on both public health and the global economy. Optimizing therapeutic options and preventing complications related to this condition are priorities. A meta-analysis by Sáenz-Ravello shows that periodontal treatment can reduce the costs of type 2 diabetes treatment and improve glycemic control [35]. These findings align with those of Seron [103]. However, the use of resolvins in the treatment of periodontitis is not yet supported by sufficient clinical evidence [53,60].

While the therapeutic potential of resolvins is promising, several challenges must be addressed before they can become standard treatments for periodontal disease and diabetes. Large-scale human clinical trials are needed to determine the optimal dosages, delivery methods, and long-term safety of resolvins, as most positive findings thus far have come from animal studies and small trials. Additionally, resolvins are naturally unstable in the body, so developing stable formulations or synthetic analogs that remain effective at the site of inflammation is essential. For periodontal disease, targeted delivery to the gums is crucial to maximize local effects, while for diabetes, achieving systemic anti-inflammatory effects without disrupting other immune functions is key.

## 8. Conclusions and Perspectives

This relationship between resolvins, periodontal disease, and type 2 diabetes highlights the critical role of inflammation in both conditions. Resolvins, by actively resolving local inflammation in the gums, can improve periodontal health, thereby potentially reducing systemic inflammatory burden. This reduction in systemic inflammation may subsequently help in improving blood sugar control and insulin sensitivity in individuals with type 2 diabetes. This interplay demonstrates how addressing a localized inflammatory condition, such as periodontal disease, can have broader effects on systemic health, opening new possibilities for treating these interconnected conditions.

In conclusion, while resolvins are not yet part of standard therapies for periodontal disease or type 2 diabetes, they represent a promising new avenue that focuses on resolving inflammation rather than merely suppressing it, offering a more holistic approach to managing chronic conditions.

## Figures and Tables

**Table 1:** The roles and mechanisms of action of resolvins in periodontal disease.

Resolvin Type	Role of Resolvin	Mechanism	Reference
RvD1	Prevents and reverses inflammation	Acts on the NF-κB signaling pathway (reverses TNF-α translocation on NF-κB p65)	[54]
	Promotes bone regeneration	Decreases osteoclast activity by reducing the RANKL/OPG ratio and promotes osteoblast differentiation	[55]
	Restores the proliferation and wound healing capacity of periodontal ligament fibroblasts (PDLFs)	Inhibits the IL-1β action on PDLFs	[56]
	Promotes the formation of calcium nodules in periodontal ligament cells (PDLCs)	Reduces the IL-1β expression in PDLCs and influence on the p38 MAPK signaling pathway	[57]
RvD2	Reduces the size of periapical lesions and improves mineralization of root canal apices	Decreases MPO (myeloperoxidase) activity and the influx of neutrophils, monocytes, and leukocytes	[58,59]
	Induces reparative dentin formation and proliferation of dental pulp stem cells (DPSCs)	Acts directly on GPR18 expressed on pulp tissue cells	[60]
	Prevents alveolar bone loss	Decreases RANKL expression and increases osteoprotegerin (OPG) expression	[61]
RvE1	Reduces bone resorption by inhibiting osteoclast growth and differentiation	Promotes both RANKL/OPG and receptor activator of NF-κB ligand/OPG ratios	[62,63,64]
	Promotes the resolution of inflammation	Reduction in CRP and IL-1β Promotes the pro-resolutive phenotype of macrophages Restores macrophage phagocytic activity	[65,66,67]
RvE2	Promotes the resolution of inflammation	Promotes the pro-resolutive phenotype of macrophages	[66]

## Data Availability

Not applicable.
